# Galactosyltransferase 4 is a major control point for glycan branching in N-linked glycosylation

**DOI:** 10.1242/jcs.151878

**Published:** 2014-12-01

**Authors:** Andrew G. McDonald, Jerrard M. Hayes, Tania Bezak, Sonia A. Głuchowska, Eoin F. J. Cosgrave, Weston B. Struwe, Corné J. M. Stroop, Han Kok, Teun van de Laar, Pauline M. Rudd, Keith F. Tipton, Gavin P. Davey

**Affiliations:** 1 School of Biochemistry and Immunology, Trinity Biomedical Sciences Institute, Trinity College Dublin, Dublin 2, Ireland; 2 National Institute for Bioprocessing Research and Training (NIBRT), Fosters Avenue, Dublin 4, Ireland; 3 Merck, Sharp & Dohme, 5340 BH Oss, The Netherlands

**Keywords:** Glycosylation, Golgi, branching, Galactosyltransferase, Chorionic gonadotropin, hCG

## Abstract

Protein N-glycosylation is a common post-translational modification that produces a complex array of branched glycan structures. The levels of branching, or antennarity, give rise to differential biological activities for single glycoproteins. However, the precise mechanism controlling the glycan branching and glycosylation network is unknown. Here, we constructed quantitative mathematical models of N-linked glycosylation that predicted new control points for glycan branching. Galactosyltransferase, which acts on *N*-acetylglucosamine residues, was unexpectedly found to control metabolic flux through the glycosylation pathway and the level of final antennarity of nascent protein produced in the Golgi network. To further investigate the biological consequences of glycan branching in nascent proteins, we glycoengineered a series of mammalian cells overexpressing human chorionic gonadotropin (hCG). We identified a mechanism in which galactosyltransferase 4 isoform regulated N-glycan branching on the nascent protein, subsequently controlling biological activity in an *in vivo* model of hCG activity. We found that galactosyltransferase 4 is a major control point for glycan branching decisions taken in the Golgi of the cell, which might ultimately control the biological activity of nascent glycoprotein.

## INTRODUCTION

N-linked glycosylation is a protein modification system present in all domains of life that is characterised by extreme complexity and a seemingly random control process. Over 60% of proteins, typically cell surface receptors and transporters, hormones, cytokines and antibodies are post-translationally modified by the addition of glycan structures as they are processed through the endoplasmic reticulum and Golgi. As the N-glycans are trimmed and re-modelled in transit through the Golgi, multiple glycoforms of a glycoprotein are generated by a process that is partially understood. The *N*-acetylglucosaminyltransferase enzymes (GnTs; GnTI, GnTII, GnTIV and GnTV) utilise UDP-*N*-acetylglucosamine (UDP-GlcNAc) as a donor substrate and transfer the GlcNAc onto the glycoform at different branching points. Flux through the hexosoamine and sugar-nucleotide pathways are known to influence this process as they modulate the levels of UDP-GlcNAc substrate available for branching (Sasai et al., 2002; Schachter, 1986). Further modification of the branched glycoforms involves galactosylation by the β-1,4-galactosyltransferases (GalTs), elongation with poly-*N*-acetyllactosamine by β-1,3-*N*-acetylglucosaminyltransferase (GnTE) and capping with sialic acid by α-2,3-sialyltransferase (SiaT). Although it is known that hexosamine flux regulates the degree of branching and number of complex N-glycans, which in turn cooperate to regulate cell proliferation and differentiation (Lau et al., 2007), the complex nature of interactions between the glycosyltransferases that construct the glycan repertoire on glycoproteins is still unresolved (Lowe and Marth, 2003).

The importance of post-translational glycosylation for the effectiveness of biotherapeutic proteins has become increasingly recognised for its role in bio-activity, serum half-life, stability and immunogenicity (Dalziel et al., 2014; Jefferis, 2009; Perlman et al., 2003; Sinclair and Elliott, 2005; Solá and Griebenow, 2009). However, controlling the glycosylation of recombinant proteins to obtain favourable glycoforms is a substantial challenge. Glycoengineering mammalian cells has led to the development of monoclonal antibodies lacking core fucosylation, resulting in a 50–100-fold higher affinity for Fcγ receptors and a concomitant increase in antibody-dependent cellular cytotoxicity (ADCC) (Ferrara et al., 2011; Ferrara et al., 2006; Iida et al., 2006; Shields et al., 2002; Shinkawa et al., 2003; Umaña et al., 1999). Increased glycan antennarity and sialic acid capping of glycoproteins prevents their premature removal from circulation by the asialoglycoprotein receptor on hepatocytes (Ashwell and Morell, 1974). In addition, it has been shown that glycoengineering of erythropoietin to produce hyper-glycosylated and sialylated forms of the protein substantially increased bioactivity and serum half-life (Egrie et al., 2003). Further understanding of the complex process controlling glycan antennarity, galactosylation and sialylation in the Golgi is required.

In this article, we describe computer-generated models and simulations of N-linked glycosylation in mammalian cells in which we identify new control points where glycosylation can be modified. These predictions were then validated in a series of host cell lines expressing a heavily glycosylated glycoprotein hormone, human chorionic gonadotropin (hCG). Our experiments identify a new feature of the regulation of kinetic flux through the glycosylation system; specifically, that galactosyltransferase activity controls N-glycan branching and identifies a means through which variant glycoforms of a single protein give rise to different effects on biological activity.

## RESULTS

### *In silico* modelling of glycan post-translational modifications

**Fig. 1.**
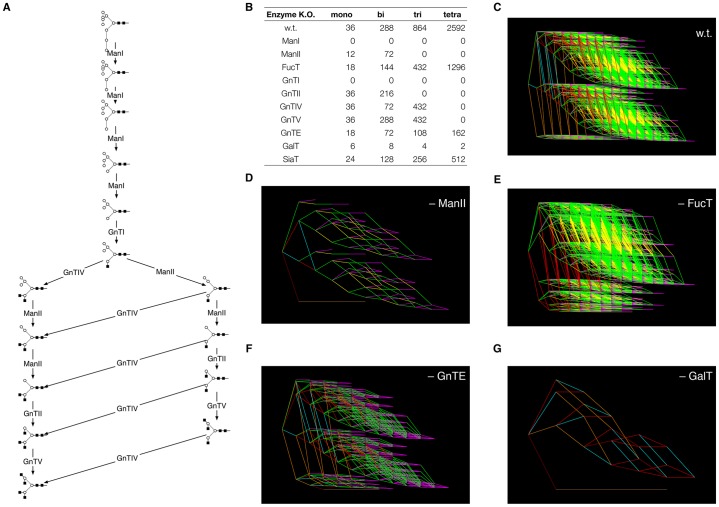
***In silico* knockouts of enzymes of N-glycosylation and their effects upon reaction network complexity.** (A) Core network of glycosylation, showing the initial stages of N-glycosylation starting with Man_9_ (M9) and further catalysed by the enzymes described in Table 1. For symbol key, see Fig. 4 inset. (B) Total numbers of mono-, bi-, tri- and tetra-antennary N-glycan structures predicted by the model for wild-type (w.t.) and individual enzyme knockouts. (C–G) Reaction network diagrams corresponding to five of the knockouts shown in B. Colour key: GnTs I, II, IV and V, red; GnTE, yellow; GalT, green; ManI, brown; ManII, orange; FucT, cyan; SiaT, magenta.

We constructed a mathematical model of N-linked glycosylation in Chinese hamster ovary (CHO) cells based on the actions of eleven enzymes that are known to be present in the CHO Golgi (see Table 1): two mannosidases, ManI and ManII; the (core) fucosyltransferase, FucT; six *N*-acetylglucosaminyltransferases, GnTI, GnTII, GnTIII, GnTIV, GnTV and GnTE; a galactosyltransferase, GalT; and a sialyltransferase, SiaT. The core network of reactions is shown in Fig. 1A. The details of the mathematical models, which were based on an earlier model by Umaña and Bailey (Umaña and Bailey, 1997) and its extension by Krambeck and Betenbaugh (Krambeck and Betenbaugh, 2005), are given in the Materials and Methods. To elucidate glycan complexity, we developed two software programs, GlycoForm and Glycologue (McDonald et al., 2010), for the display of N-glycans.

**Table 1.**
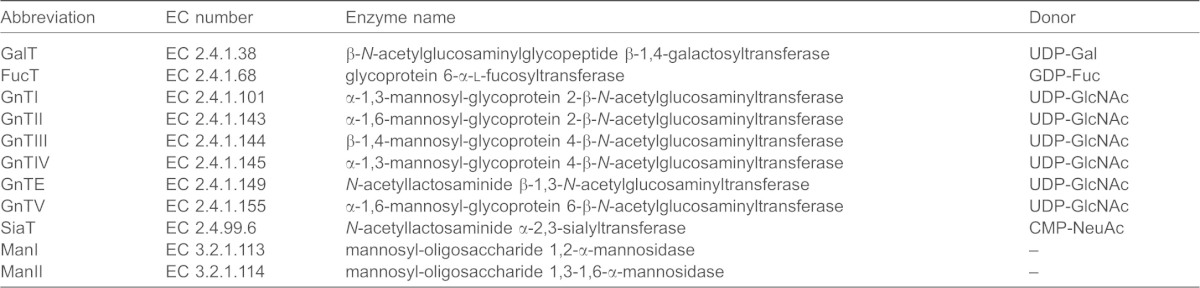
Enzymes involved in N-glycosylation in CHO cells.

Our objective was to determine the key enzymes controlling the production of tetra-antennary glycoforms. To this end, the theoretical models were used to test, *in silico*, the under- and over-expression (compared with their mean *in vivo* concentration) of each enzyme. A preliminary series of *in silico* experiments, in which each of the enzymes of CHO N-glycosylation was eliminated in turn, was used to determine the effects of individual enzyme knockouts on antennarity. The results, which are summarised in Fig. 1B–G, show that the enzymes GalT and GnTE are responsible for much of the glycoform heterogeneity.

We developed methods for the construction of mathematical models of glycosylation in thermodynamically open or closed systems, with or without the consideration of the levels of donor sugar-nucleotides, for reactions occurring within either a homogeneous single Golgi compartment or one compartmentalised into four spatial regions (or temporal phases), termed cis, medial, endo and trans-Golgi network (TGN). For simplicity, we considered a theoretical protein with a single N-glycosylation site. For open-system models it was assumed that protein substrate was introduced at a constant rate, and we assumed a mean residence time of 20 min within the whole Golgi. The results we present here are for a single-compartment and single-substrate open system, although qualitatively similar results were obtained when these restrictions were removed. Further assumptions of the model were: (i) the essential irreversibility of the reaction network; (ii) a constant supply of the donor sugar-nucleotides CMP-Neu5Ac, GDP-Fuc, UDP-Gal and UDP-GlcNAc; and (iii) constant cell and organelle volumes. Sugar-nucleotides are transferred to the Golgi by means of dedicated transporters (Hirschberg et al., 1998), which also act as antiporters, moving monophosphorylated nucleotides from the Golgi to the cytoplasm. Justification for the irreversibility criterion is provided by the existence of a nucleoside-diphosphatase (NDPase) activity within the Golgi (Hirschberg et al., 1998), which maintains the secondary products of the transferase reactions at a low level, thereby reducing the effects of product inhibition. GnTIII was omitted from the kinetic model, because it is not expressed in wild-type CHO cells (Schriebl et al., 2006; Stanley et al., 2005), and the modelling results indicated that its absence would not adversely affect the production of tetra-antennary structures.

**Fig. 2.**
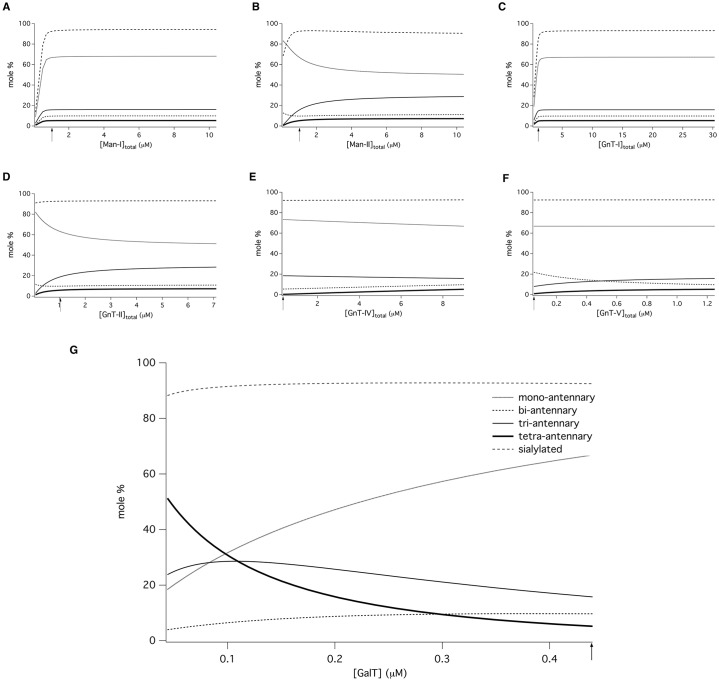
**Computational model predicts galactosyltransferase controls glycan antennarity.** *In silico* variation of local enzyme concentrations: (A) ManI; (B) ManII; (C) GnTI; (D) GnTII; (E) GnTIV; (F) GnTV; (G) GalT. Final concentrations of *N*-glycan structures of different antennarities predicted by the kinetic model are plotted as a function of the total concentration of GalT, when combined with a 25-fold overexpression of GnTIV and GnTV. The position of the arrow on each abscissa indicates the default enzyme concentration. Values on each ordinate axis are expressed as a molar percentage of the total concentration of all N-glycan forms.

Given that published data are not available for the *K*_m_ values of each enzyme for the large variety of acceptors, we used averaged values obtained from the literature (Krambeck and Betenbaugh, 2005), and substituted our own experimentally determined *K*_m_ values for the donors (nucleotide sugars), where available. In the absence of GnTIII, the number of unique N-glycans predicted by the theoretical model was 3677 in 11,541 separate reactions. Rather than follow the production of individual N-glycans, we found it convenient to create special model variables that expressed the concentrations of mono-, bi-, tri- and tetra-antennary glycoforms as molar percentages (Fig. 2). We computed the steady-state intracellular concentrations of each variable as a function of local enzyme concentration. The effects of knocking down selected enzymes individually are shown in Fig. 2A–F. Our model predicted only modest increases in tetra-antennary N-glycans when GnTs IV and V were overexpressed separately (Fig. 2E,F) or together (Fig. 2G). The most surprising, yet consistent finding, was that lowering the level of GalT was a major factor influencing the production of N-glycans of high antennarity (Fig. 2G).

A comparison of values taken from the literature indicates that the enzyme GalT (EC 2.4.1.38) has an apparent *k*_cat_/*K*_m_ value that is considerably higher than the other enzymes involved in glycosylation. Although the *K*_m_ values will depend on the nature of the acceptor (Pâquet et al., 1984), and likewise those of *k*_cat_, the quoted *k*_cat_ value for GalT is nonetheless at least four times higher than that of any other enzyme in the network. In Fig. 2G, GalT is knocked down, while the enzymes that have the greatest influence on tetra-antennarity, namely GnTIV and GnTV are overexpressed by 25-fold. This simulation revealed a trend whereby tetra-antennary production increased by ∼10-fold when the local concentration of GalT was reduced below 10% of its assumed wild-type value (0.44 µM), when all other enzyme concentrations were held constant. The results indicate that there is a flux-based competition between GalT and the other enzymes in the network. These results suggest that the overexpression of GnTIV and GnTV, in conjunction with a 90% reduction in Golgi concentrations of GalT, generates a distribution of N-glycans that is comprised of 56% tetra-antennary structures, with a high degree of sialylation (>80%). It was observed in simulation timecourses that a decrease in GalT level led to a delayed appearance of tetra-antennary forms. The results of Fig. 2G should therefore be considered the theoretical maximum. A theoretical study of N-glycosylation predicted that increases in galactosyltransferase activity can influence N-glycan microheterogeneity (Hossler et al., 2007). Our results are the first description of the effect of a decrease in such activity upon antennarity.

### Glycan branching in hCG

We next verified the model predictions in a Chinese hamster ovary (CHO) cell line engineered to express recombinant hCG (CHO-HCG). Three derivative stable cell lines were engineered to overexpress the enzymes GnTIV (CHO-GnT4), GnTV (CHO-GnT5) and GnTIV and GnTV together (CHO-GnT4,5). Overexpression of GnTIV and GnTV was detected by immunoblotting, and translocation to the Golgi was assessed by immunofluorescence microscopy. The relative amounts of GnT and GalT mRNA of each transformed cell line were determined by quantitative real-time PCR. GnTIV mRNA levels increased 15-fold and GnTV mRNA levels were increased 11-fold over that of the parent cell line. Small hairpin RNA (shRNA) constructs were used to knock down the galactosyltransferase genes GalT1 through GalT6 (Table 2).

**Table 2.**
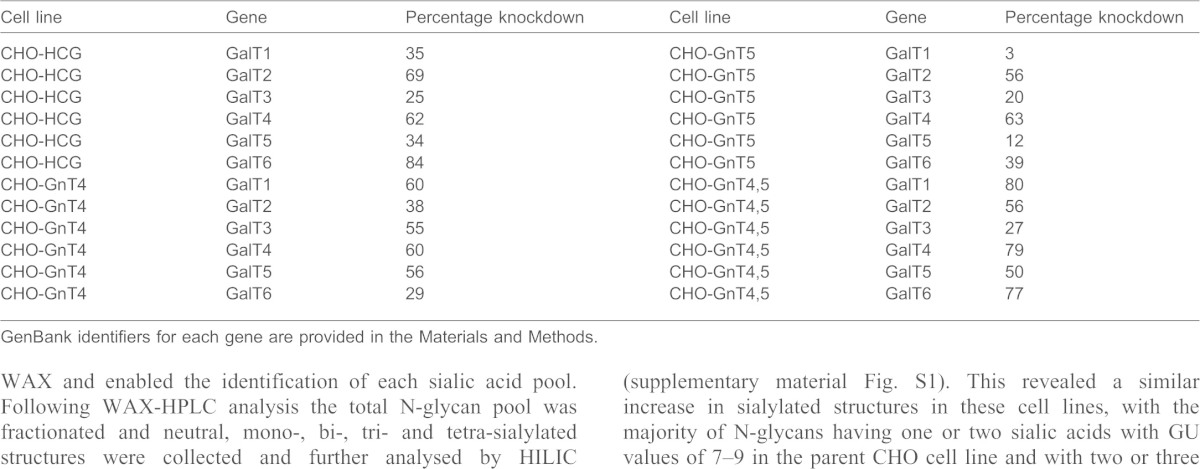
Quantitative RT-PCR results of CHO cell galactosyltransferase (GalT) knockdowns

GenBank identifiers for each gene are provided in the Materials and Methods.

The N-glycans of immunopurified hCG were removed by PNGase F digestion, fluorescently labelled with 2-aminobenzamide and analysed by hydrophilic interaction liquid chromatography (HILIC) and weak-anion exchange high-pressure liquid chromatography (WAX-HPLC), together with exoglycosidase digestions, according to Royle et al. (Royle et al., 2008). Preliminary structural assignments were made by comparison of the retention times of the N-glycans expressed in GU (glucose unit) values with those reported by the online software tool GlycoBase (http://glycobase.nibrt.ie/). Structures identified by HPLC were verified by matrix-assisted laser desorption/ionization (MALDI) mass spectrometry following permethylation.

**Fig. 3.**
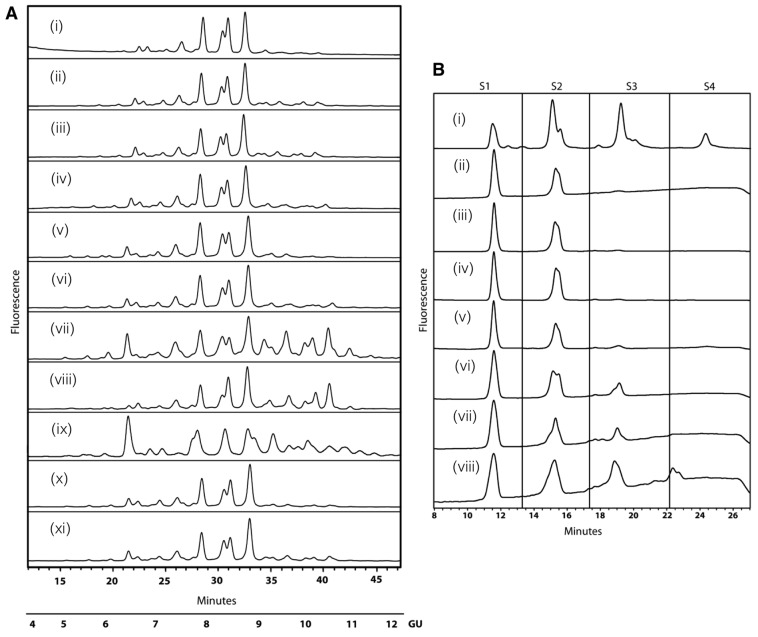
**GalT4 controls level of glycan antennarity and sialylation in N-linked glycosylation.** (A) HILIC analysis of hCG immuno-purified from engineered cell lines shows that GalT4 knockdown causes substantial changes to the glycan profiles of the substrate glycoprotein (vii, viii, ix) suggesting that GalT4 is a major flux control point for glycan branching. (i) HCG, (ii) CHO-GnT4, (iii) CHO-GnT5, (iv) CHO-GnT4-GalT1, (v) CHO-GnT4-GalT2, (vi) CHO-GnT4-GalT3, (vii) CHO-GnT4-GalT4, (viii) CHO-GnT5-GalT4, (ix) CHO-GnT4,5-GalT4, (x) CHO-GnT4-GalT5, (xi) CHO-GnT4-GalT6. (B) GalT4 knockdown increases tri-sialylated glycan structures (S3) in CHO cells. WAX-HPLC of HCG from engineered cell lines and revealed a substantial increase in tri-sialylated structures in cell lines with GalT4 knockdown (v, vi, vii). (i) Bovine fetuin standard, (ii) HCG, (iii) CHO-GnT4, (iv) CHO-GnT5, (v) CHO-GnT4,5, (vi) CHO-GnT4-GalT4, (vii) CHO-GnT5-GalT4, (viii) CHO-GnT4,5-GalT4.

Initial HILIC profiles identified seven major 2-aminobenzamide (2-AB)-labelled N-glycan peaks from hCG (Fig. 3A). No significant changes were observed in the N-glycan profiles from cells overexpressing GnTIV or GnTV alone (Fig. 3Aii,iii) or GnTIV and GnTV together or from cells with only GalT knockdowns (data not shown). Overexpression of GnTIV or GnTV in combination with knockdown of galactosyltransferase (GalT4) caused significant changes to the N-glycan profiles of hCG from these cell lines, particularly in the region of higher GU values (9–11) (Fig. 3Avii,viii). These glycosylation changes were not present for other galactosyltransferase knockdowns (Fig. 3Aiv,v,vi,x,xi). Dual overexpression of GnTIV and GnTV together with GalT4 knockdown also resulted in substantial glycosylation changes to the substrate glycoprotein. Interestingly, a major increase was observed for a glycan structure at approximately GU 6.2 (see Fig. 3Aix).

WAX-HPLC was used to analyse the degree of N-glycan sialylation of hCG and revealed a substantial increase in tri-sialylated structures in cell lines overexpressing GnTIV or GnTV alone or GnTIV and GnTV together with GalT4 knockdown (Fig. 3Bvi,vii,viii). Bovine fetuin, which contains neutral, mono, bi, tri and tetra-sialylated glycans, was used as a standard for WAX and enabled the identification of each sialic acid pool. Following WAX-HPLC analysis the total N-glycan pool was fractionated and neutral, mono-, bi-, tri- and tetra-sialylated structures were collected and further analysed by HILIC (supplementary material Fig. S1). This revealed a similar increase in sialylated structures in these cell lines, with the majority of N-glycans having one or two sialic acids with GU values of 7–9 in the parent CHO cell line and with two or three sialic acids (GU 8–10) in the engineered cell lines. In all cases, the sialic acids were in an α2,3 linkage to galactose, as shown by digestion with *Streptococcus pneumoniae* (Nan1) sialidase, a recombinant exoglycosidase specific for α-2,3-linked sialic acids.

**Fig. 4.**
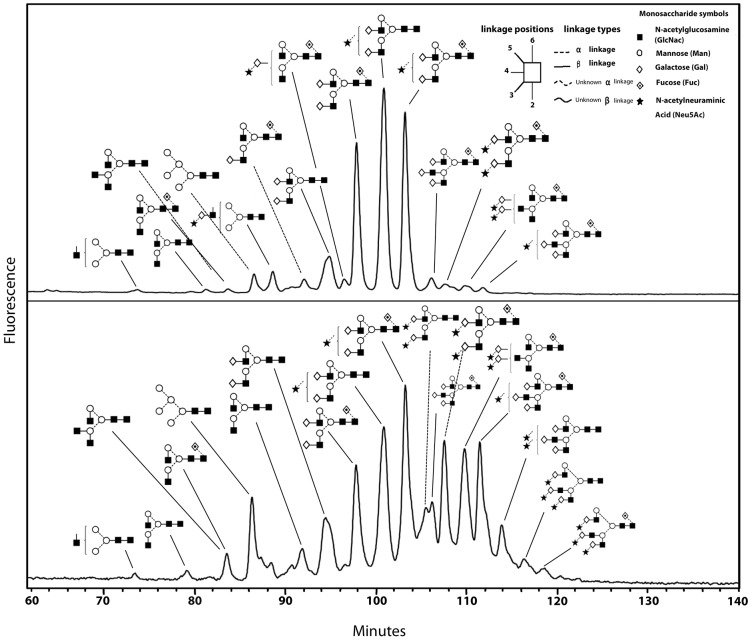
**GalT4 regulates glycan complexity in substrate glycoproteins.** Substantial increases in tri-antennary and tri-sialylated structures were observed upon GalT4 knockdown. Glycan profiles were measured by HILIC and glycan structures were determined by a combination of exoglycosidase digestions and WAX-HPLC. (A) hCG immuno-purified from CHO cells. (B) hCG immuno-purified from CHO-GnT4-GalT4 cells. Nomenclature of N-glycans is shown in the inset together with a guide to N-glycan monosaccharide symbols, linkage types and positions. For further information on the Oxford notation and nomenclature see http://glycobase.nibrt.ie/glycobase/documents/abbreviations.pdf

The HILIC profile of the undigested pool of N-glycans from the CHO-GnT4-GalT4 cell line showed a large number of overlapping peaks with high GU values, owing to the large number of differently sialylated species present (Fig. 4B). This was not seen for hCG from the parent cell line (Fig. 4A). Exoglycosidase digestions with enzymes used alone or in combinations followed by HILIC were used to elucidate the structures of the constituent monosaccharides. This was performed on the total N-glycan pool (supplementary material Fig. S2) and on the WAX fractionated samples. This is a two-dimensional glycan analysis using WAX in the first dimension and HILIC in the second dimension as a way to deconvolute complex glycan pools for structural assignments. The subsequent analysis revealed that the majority of N-glycans of hCG expressed in CHO cells were predominantly complex bi-antennary glycans, A2G(4)2, F(6)A2G(4)2, A2G(4)2S(3)1 and F(6)A2G(4)1S(3)1 [as presented in Oxford notation, for more information, see http://glycobase.nibrt.ie/glycobase/documents/abbreviations.pdf], comprising ∼8.5%, 21.5%, 26% and 23.5% of the total N-glycans, respectively (Fig. 4A). Large decreases in these bi-antennary glycans with concomitant increases in tri-antennary structures, particularly F(6)A3G(4)3, F(6)A3G(4)2S(3)2, F(6)A3G(4)3S(3)1, A3G3S2 were present in the CHO-GnT4-GalT4 cell line (Fig. 4B). Results of the N-glycan analysis of hCG are summarised in Table 3 and the proposed structures are shown in supplementary material Table S1. Interestingly, substantial increases were also observed in the N-glycan Man_5_ [Man_5_ refers to the core N-glycan plus an additional two mannose residues, see http://glycobase.nibrt.ie/glycobase/documents/abbreviations.pdf], which increased ∼10-fold, from 0.4% to 4%, in this cell line. Substantial decreases in bi-antennary structures and increases in tri-antennary structures were also observed in the CHO-GnT5-GalT4 cell line. A similar effect was observed when both GnTIV and GnTV were co-overexpressed with GalT4 knockdown and a further substantial increase was seen in Man_5_, which increased to ∼15% of the total N-glycans of hCG from this cell line. Tetra-antennary structures in the hCG cell line were 0.1% of the total glycans; however, they increased ∼10–14 fold in the engineered cell lines, displaying similarity to the trend observed in the modelling experiments (Fig. 2G). Tandem mass spectrometry (MS/MS) was used to verify the structures identified by HPLC (see supplementary material Fig. S3). In addition to major changes in antennarity, antennary galactosylation and sialylation were increased substantially in the engineered cell lines. Approximately 70% of structures were found to be core fucosylated. All galactose residues were found to be in β-1,4 linkages to GlcNAc residues, as assessed by digestion with *Streptococcus pneumoniae* β-galactosidase, an enzyme that specifically cleaves galactose linked to *N*-acetylglucosamine in a β(1,4) position.

**Table 3.**
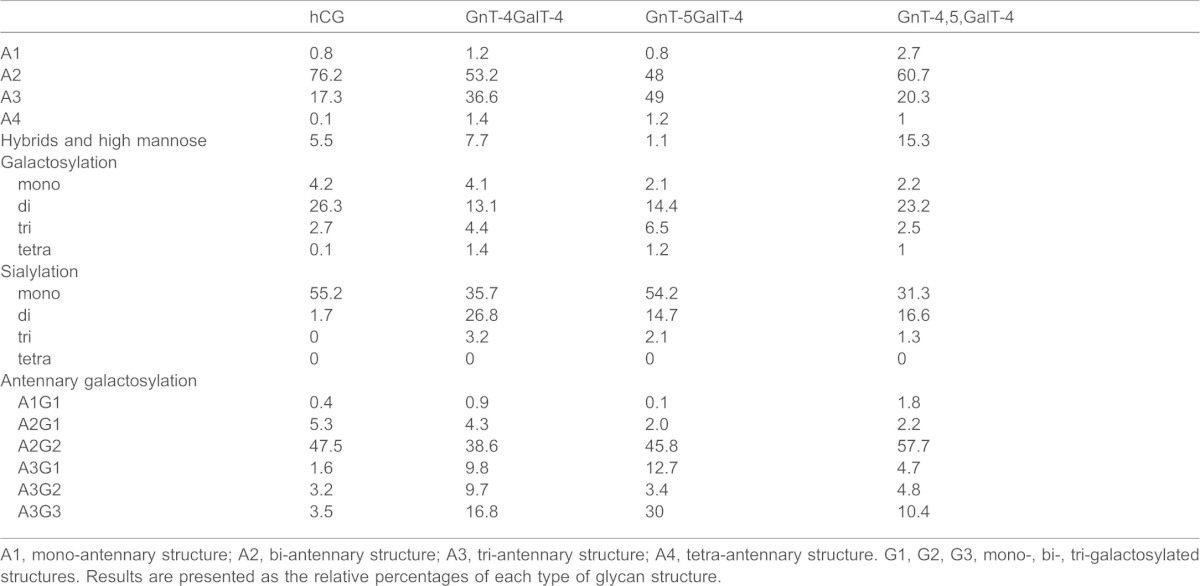
Results of N-glycan analysis of hCG and summary of the effects of overexpression of GnTIV, GnTV and knockdown of GalT4 on the glycosylation of the substrate hCG

A1, mono-antennary structure; A2, bi-antennary structure; A3, tri-antennary structure; A4, tetra-antennary structure. G1, G2, G3, mono-, bi-, tri-galactosylated structures. Results are presented as the relative percentages of each type of glycan structure.

### *In vivo* hCG activities

The biological activity of hCG was measured using a seminal vesicle weight assay. The basis of this assay is that the hormone increases the weight of the seminal vesicles of immature rats which are then excised and weighed and values are expressed as a measure of hormone activity. Engineered hCG from parent CHO and two stable engineered cell lines was injected subcutaneously into immature male rats. The potency of hCG expressed in parent CHO cells was found to be 16,200 IU/ml. The potency of hCG from GalT4-knockdown cells overexpressing GnT4 was 11,300 IU/ml, whereas hCG from GalT4-knockdown cells overexpressing both GnTIV and GnTV had a potency of 11,400 IU/ml, reflecting a reduction in efficacy of ∼30% compared to control.

## DISCUSSION

The aims of this study were to comprehend and model the complex N-glycosylation system in mammalian cells, and to identify unique control points governing glycan branching and subsequent biological efficacy. Our study demonstrates a novel means by which the antennarity of N-glycans on protein expressed in CHO cell lines can be modified and increased. Our approach was guided by computer simulations of N-glycosylation in CHO cells and was based upon a principle of kinetic competition for glycoprotein substrates. The simulations were, in turn, refined by the subsequent experimental results. A major finding of these simulations was that translational suppression of galactosyltransferase activity enhances the effects of the N-glycosyltransferases that are responsible for the formation of high-antennary complex N-glycans. The results obtained from the mathematical model depend on the relative values of the parameters, rather than their absolute values. Initially, the *in silico* knockouts (Fig. 1B–G) showed that tri- and tetra-antennary structures could be formed in the absence of β-1,4-galactosyltransferase activity, despite their lack of galactose or sialic acid residues. This prompted an investigation into the regulation of GalT activity. Subsequently, our *in-vitro* experiments were based on the computer simulation results, which showed a correlation between downregulation of GalT activity and an increase in both tri- and tetra-antennary structures (Fig. 2G). *In silico* variation of the concentrations of GnTE, FucT and SiaT at 10-fold above and below their default values had little or no effect upon antennarity in the steady state.

Our experimental approach was to test the computer-simulated results in a CHO cell line engineered to overexpress the glycoprotein hormone hCG. Sugar chains of naturally occurring hCG, and hCG purified from CHO cells are mainly composed of bi-antennary complex N-glycans (Kessler et al., 1979; Weisshaar et al., 1991). Our experiments demonstrate that a decrease in galactosyltransferase activity is required, possibly through a competitive effect with GnTIV and GnTV for access to the antennary GlcNAc residues. Interestingly, it is primarily the reduction in GalT4 activity that results in substantial 2–3-fold and 10–14-fold increases in the relative levels of tri- and tetra-antennary N-glycan structures, respectively (Table 3). This glycoengineering approach delivers hCG protein in which the majority of glycans are of the tri-antennary structure. The 10–14-fold increase in tetra-antennary glycans in the engineered cell lines reflects a similar 10-fold increase observed in the mathematical modelling experiments. Large differences in sialylation were also observed, although we found that the higher antennary glycans were not fully sialylated, possibly owing to the insufficient α-2,3-sialyltransferase activity in CHO cells (Baranski et al., 1990; Fukuta et al., 2000). Substantial glycosylation changes also occurred when GnTIV and GnTV were co-overexpressed when GalT4 was knocked down (Fig. 3Aix), with observed increases in tri- and tetra-antennary structures, although the effect was not as marked as when each GnT was individually overexpressed. Although overexpression of GnTV might have been expected to give more substantial increases in tetra-antennary structures, their absence is likely due to the protein backbone structure of hCG, as this is considered to be the determining factor for the addition of highly branched sugar chains to glycoproteins (Lau et al., 2007; Lis and Sharon, 1993; Stanley, 2007; Tulsiani et al., 1982; Ujita et al., 1999). Intriguingly, the dual-enzyme overexpression system gave an increase in oligomannose structures that were almost entirely in the form of Man_5_, a result that could be attributed to a reduction in the flux through GnTI, as further simulation results were able to confirm. A reduction in ManII activity would not be expected to give the same result because this enzyme has a marked preference for Man_5_GlcNAc (Lee et al., 2001). Whereas a reduction in the level of GalT might also be expected to reduce the extent of sialylation, as is the case with the galactosylation-deficient CHO Lec8 mutant cell line (Stanley, 1989), such a decline was only noticeable in the case of dual-overexpression and might be ascribed to the underprocessing of Man_5_. An interesting observation was the effect of GalT4 on the galactosylation of N-glycans, as this isoform is usually associated with the galactosylation of mucin core-2 O-glycans (Lee et al., 2001). This finding may provide indirect confirmation of an earlier report of full galactosylation of tetra-antennary N-glycans in the absence of any GalTI activity (Hossler et al., 2007). Our data suggest that GalT4 is the dominant transferase in galactosylation of N-glycans in CHO cells.

The enzyme GnTE is responsible, in combinatorial fashion, for much of the potential variety of N-glycan structures, owing to its action in conjunction with the other glycosylation enzymes. Although one recent study has provided evidence of complex extended-chain N-glycan structures in CHO cells (North et al., 2010), our analysis did not discover any antennae containing *N*-acetyllactosamine repeating units. This was a prediction of the model under conditions where the levels of nucleotide sugar-donors are saturating because this enzyme has the lowest *k*_cat_/*K*_m_ of the enzymes of N-glycosylation. Therefore, much higher expression levels would be required before such structures would be detected in fluorescent-labelling experiments.

Recent evidence has shown that several Golgi enzymes are capable of forming homodimers and heterodimers (Hassinen et al., 2010; Schoberer et al., 2013), supporting the ‘kin recognition’ model of the retention of functionally related Golgi enzymes (Nilsson et al., 1993). In conjunction with these findings, our results might be an indication that GalT4 and GnTs IV and V colocate in the same region of the Golgi. To our knowledge, the effects of cooperativity on the kinetics of glycosylation have not yet been determined. The effects of enzyme knockdown and overexpression on the localisation of the enzymes, as well as their kinetics, is a subject deserving of future study.

The carbohydrates of glycoproteins are crucial in mediating their biological function, serum retention and interaction with receptors and binding partners. A classic example of this is immunoglobulin G. The N-linked glycans attached to the canonical asparagine 297 residues in both heavy chains of the antibody molecule determine the binding to Fcγ receptors and therefore dictate the immunological response. In addition, individual monosaccharide residues of IgG specify the particular immune response induced by IgG: for example, IgG lacking galactose (IgG G0) residues can interact with mannose-binding lectin (MBL) to activate complement (Malhotra et al., 1995); IgG galactosylation is increased in pregnant women and is involved in placental transport (Kibe et al., 1996); sialylation of IVIG is involved in anti-inflammatory effects (Kaneko et al., 2006); and loss of the α(1–6) core fucose results in increased binding to FcγRIIIa and a concomitant increase in ADCC (Okazaki et al., 2004). What we observed, in the case of hCG with increasing the glycan antennarity and sialylation of the hormone, was a consistent decrease in the biological effect *in vivo*, as measured in seminal vesicle weight assays. The molecular basis for this decrease in biological activity might be due to the larger and bulkier glycans found on glycoengineered hCG and decreased binding of the hormone to the luteinising hormone/choriogonadotropin receptor (LHCGR) of the ovary and testis.

**Fig. 5.**
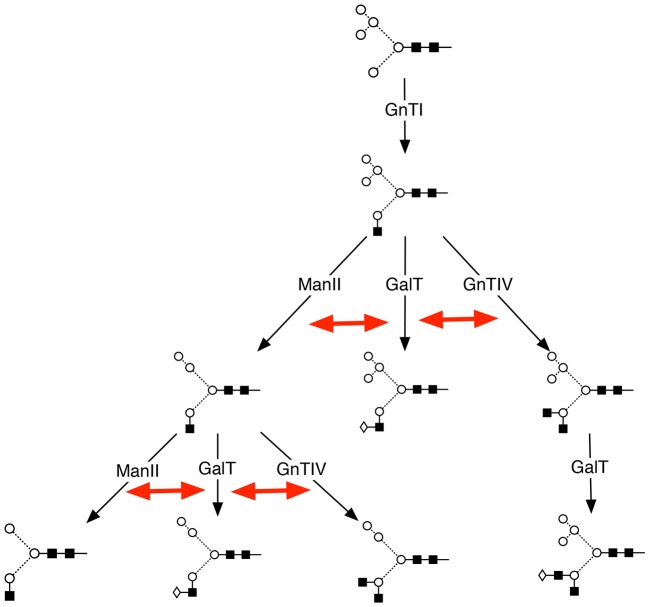
**Galactosyltransferase controls glycan branching by a process of internal kinetic competition among the enzymes of N-glycosylation.** Starting with the Man_5_, and following the action of the enzyme GnTI, the enzymes GnTIV, ManII and GalT act in parallel. At the steps indicated by the double-headed red arrows, GalT acts in competition with those enzymes leading to oligosaccharide structures of higher antennarity (branching level). Similar competition exists between the subsequent activities of GnTII, GnTV and GalT (not shown).

In summary, our findings demonstrate the predictive value of the computer model and the identification of galactosyltransferase as a major control point for N-glycan branching (Fig. 5). The results show that cells have in-built mechanisms for glycoengineering proteins that subsequently affect therapeutic efficacy. The combination of computer modelling and bioengineering with recent advances in sequencing of the CHO genome (Xu et al., 2011) will be a powerful toolset for elucidating further aspects of the N-linked glycosylation network and for improving the development of biotherapeutics.

## MATERIALS AND METHODS

### Chemicals, enzymes and reagents

Restriction enzymes were purchased from Roche Diagnostics Ltd (West Sussex, UK). PNGaseF and exoglycosidase enzymes were from Prozyme (Hayward, Ca. 94545, USA) Nuclease-free water and TRI reagent were from Ambion Inc. (Austin, TX, USA). CD-CHO chemically defined medium, L-glutamine, HT supplement, Dulbecco's modified Eagle's medium (DMEM) containing 4500 mg/l glucose, MEM non-essential amino acids, the SuperScript III First-Strand Synthesis System for RT-PCR, pCR4Blunt-TOPO vector, One Shot chemically competent TOP10 *E. coli*, ViraPower™ Lentiviral Packaging Mix, Lipofectamine™ 2000 Transfection Reagent, heat inactivated fetal bovine serum, trypsin and Opti-MEM I reduced serum medium were obtained from Invitrogen (Dun Laoghaire, Ireland). *Pfu* DNA polymerase, 10 mM dNTPs, GoTaq Flexi polymerase were from Promega Ltd (Southampton, UK). The Qiagen Plasmid Mini Preparation kit, the EndoFree Plasmid Purification kit, the QIAquick PCR purification kit, the QIAquick gel extraction kit, the RNeasy Mini Kit and the DNase set were bought from Qiagen Inc. (Dorking, Surrey, UK). Oligonucleotides and sequencing were provided by Eurofins MWG Operon (Ebersberg, Germany). The pRNAT-U6.2/Lenti constructs were purchased from GenScript USA Inc. (Piscataway, NJ, USA). The AffinityScript QPCR cDNA Synthesis Kit and the Brilliant® II SYBR® Green QPCR Master Mix were from Agilent Technologies (Cork, Ireland).

### Mathematical models of glycosylation

Models of the N-glycosylation reactions of CHO cells were developed as systems of ordinary differential equations. The fullest expression of the model is thermodynamically open and includes compartmentation of the Golgi into four regions, cis, medial, endo and trans-Golgi network. As with the earlier model of Umaña and Bailey (Umaña and Bailey, 1997), and its subsequent extension (Krambeck and Betenbaugh, 2005), an initial glycoform is assumed to enter the system at a constant rate, represented by the zero-order rate constant, *q_p_*. Glycosylated proteins leave the system at a rate proportional to their concentrations, with a first-order rate constant *k*_G_, defined as the reciprocal of the mean residence time in the Golgi.

Letting *x_i_* denote the concentration of the *i*-th N-glycan, *X_i_*, and *f_i_* the net rate of change of *x_i_* through the action of the enzymes of N-glycosylation (see Table 1), the equations of the model take the general form:
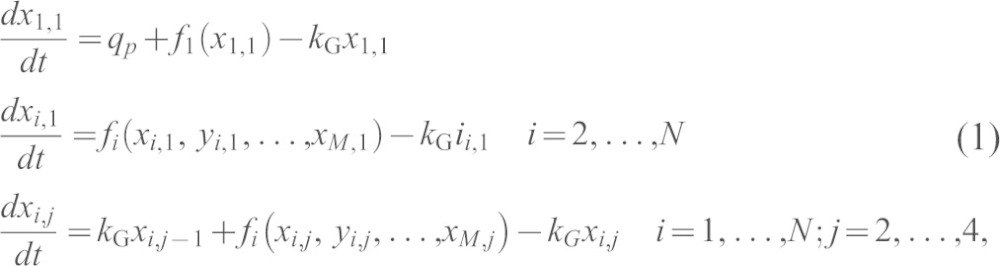
where *x_i,j_* denotes the concentration of N-glycan *X_i_* in Golgi compartment number *j*; *f_i_* is the overall rate law describing the net rate of change of *X_i_* through enzyme action; *N* is the total number of structures predicted by the model; *M* is the number of N-glycans *Y_i_* leading directly to the formation of *X_i_*.

The five primary assumptions of model are: (1) rates of influx (*q_p_*) and efflux (*k*_G_) are constant; (2) cell and organelle (Golgi) volumes are constant; (3) concentrations *x_i_* are those of a glycoprotein with a single N-linked glycosylation site; (4) all reactions are irreversible; and (5) compartments are spatially homogeneous.

The functions *f_i_* were sums of enzyme-kinetic rate laws. A single-substrate Michaelis–Menten function was used to describe the kinetics of the mannosidases ManI and ManII:
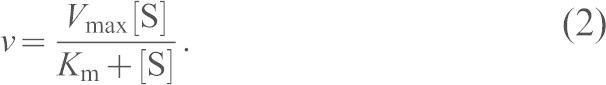
For reactions catalysed by the glycosyltransferases and GalT, a random-order mechanism was used, which assumed irreversibility according to assumption number 4. For the reactions of the form Ax + B → A + Bx, where Ax is the donor molecule and B is the glycoprotein acceptor, the rate of formation of products is given by:
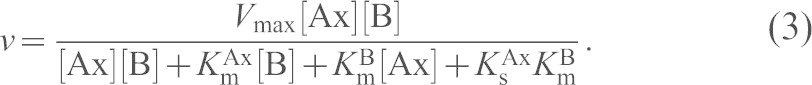
*K*_m_ values for each donor were obtained from values published in the literature. Given that published data on substrate–enzyme dissociation constants (*K*_s_ values) are scarce, it was assumed that 
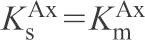
 for all Ax. The irreversibility criterion is justified by the presence of nucleoside-diphosphatase activity (EC 3.6.1.6) in Golgi, which would tend to keep the concentrations of UDP and other nucleoside diphosphates at low levels and would additionally minimise the effects of product inhibition. The overall rates of change of sugar-nucleotide donor Ax (UDP-GlcNAc, UDP-Gal, GDP-Fuc and CMP-NeuAc) were incorporated into the model according to:
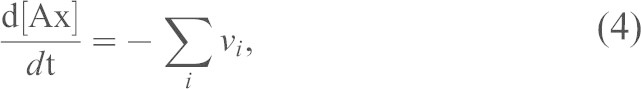
for each reaction *i* in which a molecule of Ax is converted into A, and where each of the *v_i_* is of the form of equation (2) or (3).

As a first approximation, the enzymes of N-glycosylation were distributed according to data obtained from a HeLa cell line (Rabouille et al., 1995). In the absence of specific localisation data, the other enzymes, FucT, GnTs II–V and GnTE, were distributed equally between compartments. In simulations, the percentages were normalised relative to the compartment with the highest value; the normalised values were then applied as factors to the *V*_max_ in each rate equation of the model.

The differential equations were integrated in Berkeley Madonna (http://www.berkeleymadonna.com/). Steady-state concentrations were computed independently – first in Madonna, by integrating for 100 min of simulation time and recording the final concentrations, and, second, in Mathematica (Wolfram Research, Inc.) by means of the FindRoot[] function.

### Cell culture

A CHO cell line (CHO-HCG) overexpressing recombinant hCG was provided by Organon/Merck (Oss, The Netherlands). CHO-HCG cells were cultured between at 0.2×10^6^ and 1×10^6^ cells/ml in 250 ml sterile Erlenmeyer shake flasks (Corning Life Sciences) on an orbital shaker at 200 rpm in a humidified atmosphere at 37°C with 5% CO_2_. Cell culture supernatant from CHO-HCG cells was used as a source of recombinant HCG for glycan analysis and was prepared by harvesting cells at 130 ***g*** for 10 min at 4°C.

### Immunopurification of hCG

Cell-free supernatants from stable cell lines were prepared by centrifugation at 300 ***g*** for 10 min and 4°C and then filtered through a 500 ml Filtropur V 25 filtration unit of pore size 0.2 µm (Sarstedt) and diluted 1∶1 with PBS-methionine buffer (4.1 mM Na_2_HPO_4_•2H_2_O, 1.76 mM KH_2_PO_4_, 0.13 M NaCl, 0.1 mg/ml L-methionine, pH 7.2). Columns were loaded with 1 ml of NHS-Sepharose 4 FF resin conjugated to the monoclonal antibody against β-hCG (number 119A) which was a gift from NV Organon (Oss, The Netherlands). All purification steps were carried out at 4°C under gravity flow. Columns were equilibrated with 50 ml of PBS-methionine buffer. Diluted cell-free supernatants were loaded onto the immune-affinity columns at a flow rate of 1 ml/min. Columns were washed with 50 ml of wash buffer (4.1 mM Na_2_HPO_4_•2H_2_O, 1.76 mM KH_2_PO_4_, 2 M NaCl, 0.1 mg/ml L-methionine, pH 7.2), followed by 50 ml of PBS-methionine buffer at a flow rate of 2 ml/min. Purified hCG was eluted with 45 ml of elution buffer (10 mM ammonium acetate, 0.1 mg/ml L-methionine, pH 2.7) at a flow rate of 0.5 ml/min. The eluted fractions were immediately neutralised to a pH between 7 and 8 with 25% ammonia and concentrated to ∼1 mg/ml using 10-kDa Amicon Ultra-15 Millipore columns and then desalted using PD-10 columns (Amersham Biosciences). The protein concentrations of the hCG protein samples were determined by the method of Bradford (Bradford, 1976) and standardised with BSA. The hCG samples were snap frozen in liquid nitrogen in 60 µg aliquots and freeze dried.

### SDS-PAGE analysis of hCG

SDS-PAGE was performed as described by Laemmli (Laemmli, 1970) using a 5% (w/v) polyacrylamide stacking gel and 12% (w/v) polyacrylamide resolving gel. The hCG samples were diluted with 5 µl of 4× sample loading buffer, as previously described (Laemmli, 1970), and denatured at 95°C for 3 min and samples (10 µg) per well were loaded. Electrophoresis was performed at 200 V for ∼1 h at room temperature in a Mini-Protean II Cell apparatus (BioRad) filled with running buffer (2.5 g Tris base, 14.4 g glycine and 1 g SDS dissolved in 500 m l of double-distilled H_2_O, pH 8.3). Proteins resolved by SDS-PAGE were visualised by staining with Coomassie Blue stain (500 ml methanol, 500 ml H_2_O, 100 ml acetic acid, and 2 g Coomassie Brilliant Blue R-250) for at least 30 min on an orbital shaker at room temperature. The gel was destained for ∼3 h in 10% methanol 10% acetic acid, 80% MiliQ H_2_O). For each sample, the three hCG subunits were excised from the SDS-PAGE gel and stored at −80°C.

### Sequence information

The mRNA sequences of the *Cricetulus griseus* (Chinese hamster) GnT and GalT genes were obtained from the National Centre for Biotechnology Information (NCBI; http://www.ncbi.nlm.nih.gov). The GenBank accession numbers of GnTI, GnTIII, GnTV and GalTs 1, 2, 3, 4, 5 and 6 are U65791, AY598727, U62587, AF318896, AY117536, AY117537, AY117538, AY117539 and AF506839, respectively. Sequence alignments of Chinese hamster, *Homo sapiens* (human), *Mus musculus* (mouse) and *Rattus norvegicus* (rat) revealed that Chinese hamster GnTs are highly homologous to mouse and rat GnTs (89 to 92% identical). Furthermore, the percentage sequence identities of GnT genes within each species were less than 7%. Thus, specific RT-PCR primers were designed based on mouse and rat GnTII and GnTIV gene sequences, and used to clone GnTII and GnTIV, respectively, from total RNA extracted from CHO cells. It should be noted that these experiments were performed prior to release of the CHO-K1 genomic sequence (Xu et al., 2011).

### Generation and sequencing of CHO GnTII, GnTIV and GnTV cDNAs

RNA was isolated from CHO cells using TRI reagent (Ambion) according to the manufacturer's instructions. CHO RNA (4 µg) was reverse transcribed into first strand cDNA using the SuperScript III First-Strand Synthesis System for RT-PCR (Invitrogen) according to the manufacturer's protocol. An oligo(dT) primer was used to generate GnTII and GnTV first-strand cDNA. A gene specific GnTIV primer was used to generate the first stand cDNA for GnTIV. RT-PCR primer sequences used for the amplification of each gene were: GnTII forward: 5′-TGGAGACCATGAGGTTCCGCATCTAC-3′; GnTII reverse: 5′-TCACTGCAGTCTTCTATAAC-3′; GnTIV forward: 5′-ATGAGGCTCCGCAATGGCACCTTCC-3′; GnTIV reverse 5′-CTAGTCGGCCTTTTTCAGAAAGATC-3′; GnTV forward: 5′-ATGGCTTTCTTTACTCCCTGGAAGTTGTCC-3′; GnTV reverse: 5′-CTATAGGCAGTCTTTGCATAGGGCCACTTG-3′. PCR cycling conditions were as follows: GnTII cDNA was denatured at 95°C for 2 min. This was followed by 30 cycles of denaturation at 95°C for 1 min, annealing at 49°C for 30 s and extension at 72°C for 3 min. A final extension at 72°C for 7 min completed the PCR. GnTIV cDNA was amplified using the same PCR conditions as GnTII, except the annealing temperature was 45°C. GnTV was denatured at 95°C for 2 min, underwent 35 cycles of 95°C for 1 min, 62°C for 30 s, 72°C for 4.5 min and a final cycle of 72°C for 7 min. The proofreading Pfu DNA polymerase (Promega) and 1 µM each of forward and reverse primer were used to amplify 2 µl of first strand cDNA. Following amplification GnT II, IV or V, cDNAs were resolved on a 1% (w/v) agarose gel, as described previously (Sambrook et al., 1989). A QIAquick gel extraction kit (Qiagen) was used to extract and purify the cDNAs from the agarose gel, followed by cloning into the pCR4Blunt-TOPO vector (Invitrogen) according to the manufacturer's instructions. The resulting constructs were transformed into and propagated in One Shot chemically competent *E. coli* TOP10 cells by heat-shock. Direct colony PCR using GoTaq Flexi DNA polymerase (Promega) was used to screen for positive transformants using 2 µl of a 200-µl culture inoculated with single colonies as template for PCR. The same primer sequences (described above) (each 0.4 µM final) were used to screen for positive GnT transformants. PCR products were analysed by agarose gel electrophoresis and positive transformants were selected, cultured and purified using a Qiagen Plasmid Mini Preparation kit (Qiagen). DNA inserts were sequenced by Eurofins MWG Operon using the sequencing primers T3, T7, M13 uni (−21) and M13 rev (−29).

### Generation of stable CHO cell lines overexpressing GnTIV and GnTV

CHO GnTIV and GnTV were reverse transcribed from mRNA as described above and cloned into pBluescript SK+ (Stratagene) followed by sub-cloning into the mammalian expression vector pcDNA6/V5-His (Invitrogen) to create the plasmids pcDNA6GnT4 and pcDNA6GnT5 for high level, stable expression in CHO-HCG cells. Both genes were cloned in-frame with a C-terminal V5 epitope. The sequences of the forward primers used were: 5′-CATAAGCTTGCCACCATGAGGCTCCGCAATGGC-3′ and 5′-CTAAGCTTGCCACCATGGCTTTCTTTACTC-3′ for GnTIV and GnTV respectively. The sequences of the reverse primers used were: 5′-CATGCGGCCGCTGTCGGCCTTTTTCAGAAAGATC-3′ and 5′-GTCCGCGGATATAGGCAGTCTTTGCATAG-3′ for GnTIV and GnTV respectively. Prior to transfection, the pcDNA6GnT4 and pcDNA6GnT5 plasmids were linearised by digestion with the restriction enzyme Sca-1 and purified by phenol-chloroform extraction. CHO-HCG cells (1×10^6^) were transfected with the linearised plasmids using Amaxa cell line Nucleofector Kit V according to the manufacturer's instructions. Transfected cells were cultured in CD-CHO medium plus supplements for 24 h and then selected for GnT-IV/V overexpression using the antibiotic blasticidin (5 µg/ml) in 96-well plates with ∼1000 cells seeded per well. Overexpression of GnTIV or GnTV was detected by immunoblotting using mouse monoclonal antibodies directed against the C-terminal V5 epitope. Briefly, following 3–4 weeks of blasticidin selection, cells (1×10^4^) were harvested by centrifugation at 0.8 ***g*** for 5 min at 4°C, washed with PBS and resuspended in RIPA buffer (25 mM Tris-HCl pH 7.6, 150 mM NaCl, 1% HILIC-40, 1% sodium deoxycholate, 0.1% SDS). Cells were gently agitated at 4°C for 30 min and cell debris and unlysed cells were removed by centrifugation at 120 ***g*** for 5 min at 4°C. 10 µg of total protein was subjected to SDS-PAGE as described by Laemmli (Laemmli, 1970) and following separation, proteins were transferred to PVDF membrane (Millipore) and probed with mouse monoclonal anti-V5 antibodies (1:5000 dilution). A secondary goat anti-mouse horseradish peroxidase (HRP)-conjugated antibody (1:20,000) was used to detect the primary anti-V5 antibody and proteins were visualised by enzyme-linked chemiluminescence using Millipore ECL reagents.

### Immunofluorescence microscopy

The intracellular location of overexpressed GnTIV and GnTV was determined by immuno-fluorescence microscopy using mouse monoclonal antibodies directed against the C-terminal V5 epitope. CHO cell samples were prepared by centrifugation at 800 ***g*** for 5 min at 18°C and washed with PBS. Cells were resuspended and fixed with paraformaldehyde (6%) in PBS (1∶1). Following washing in PBS the cell suspension (20 µl) was placed on a poly-L-lysine coated slide and incubated at room temperature for 15 min. Cells were incubated with the primary V5 antibody (1∶1000) in PBS containing Tween-20 (0.01%), BSA (5%) and sodium azide (0.1%) for 1 h at room temperature and washed in PBS. The cells were then incubated with 1∶1000 dilution of a secondary goat Alexa-Fluor-546-conjugated anti-mouse-IgG antibody (Invitrogen). Slides were mounted with Vectashield containing DAPI stain (Vector Laboratories Ltd) and viewed using a Zeiss Axios fluorescent microscope. The Golgi marker antibody GM130 (Abcam) was used to locate the Golgi membrane within CHO cells.

### Generation of stable shRNA knockdown cell lines

Recombinant lentivirus was produced in HEK293FT cells using 9 µg of ViraPower™ Lentiviral Packaging Mix (Invitrogen), 3 µg of endotoxin-free lentiviral construct and 36 µl of Lipofectamine™ 2000 Transfection Reagent (Invitrogen) according to the manufacturer's instructions. Viral supernatants were filtered through a Millex-HV 0.45 µm PVDF filter (Millipore) and stored as 0.5 ml aliquots at −80°C. CHO cells were transduced with a 0.5 ml aliquot of viral supernatant in the presence of 6 µg/ml polybrene (Millipore) according to the manufacturer's instructions. Cells stably expressing the shRNA knockdown constructs (Genscript, sequences are available upon request) were selected by fluorescent activated cell sorting (FACS) based on the co-expression of coral green fluorescent protein (cGFP), contained within the PRNAT.U6 lentiviral vector to achieve 98–100% GFP positive cells. The stable cell lines were expanded and supernatants were collected and stored at −80°C.

### Real-time quantitative PCR

RNA was extracted from the stable cell lines and purified using an RNeasy mini kit (Qiagen) and RNase free DNase Set (Qiagen). Each RNA pellet was resuspended in 40 µl RNase-free *d*H_2_O (Ambion) and stored at −80°C. The cDNAs were generated from the RNA templates using an AffinityScript QPCR cDNA Synthesis Kit (Agilent Technologies). Real-time quantitative PCR was carried out using a Brilliant® II SYBR® Green QPCR kit (Agilent Technologies) and a Stratagene MxPro-Mx3000P QPCR machine (Agilent Technologies). The cycling parameters were as follows. Segment 1: 1 cycle of 95°C for 10 min. Segment 2 (association curve): 40 cycles of 95°C for 30 s, 50°C for 1 min and 72°C for 30 s. Segment 3 (dissociation curve): 1 cycle of 95°C for 1 min, 55°C for 30 s and 95°C for 30 s. Real-time quantitative PCR primer sets were designed using Beacon Designer 7 software (Premier Biosoft International) and synthesised by Eurofins MWG Operon. The optimum forward and reverse primer concentrations were determined using 200 ng of wild type CHO7J RNA as template and all nine possible mixes of 200, 400 and 600 nM forward primer with 200, 400 and 600 nM reverse primer. The optimum RNA template amount and percentage efficiency of each primer set were determined from a standard curve using cDNA amounts equivalent to 999 ng, 333 ng, 111 ng, 37 ng, 12.3 ng, 4.1 ng and 1.37 ng of iHILICut RNA template. The optimum forward and reverse primer concentrations were used in the real-time quantitative PCR reactions and reactions were set up in triplicate. The percentage gene knockdowns were determined using a β-actin primer set as normalizer, wild-type template as calibrator and knockdown samples as unknown. The equivalent of 90 ng of iHILICut RNA was used as template. Reactions were set up in triplicate and the program comparative quantitation (calibrator) was run. When the program was complete, Rel. Quant. to Cal. (dR) was chosen from the results menu to view the percentage of mRNA remaining.

### Glycan analysis of hCG

N-glycans were released from hCG and analysed by the method described by Royle et al. (Royle et al., 2006) with a number of modifications. Briefly, gel slices containing hCG were washed with 20 mM NaHCO_3_, pH 7, dried in a vacuum centrifuge and rehydrated in 20 µl NaHCO_3_ (30 mM), pH 7 containing 100 mU protein-N-glycosidase F (PNGaseF). The gel slices were incubated at 37°C overnight. The PNGaseF-released glycans were eluted with three alternating washes of miliQ water and acetonitrile and evaporated to dryness in a vacuum centrifuge. Dried glycans were reduced and alkalylated by incubation in formic acid (1% v/v) at 37°C for 1 h and dried as above. Glycans were then fluorescently labelled with 2-aminobenzamide (2-AB) using a labelling kit (Ludger Ltd). Excess 2-AB label was removed using Phytip normal-phase resin columns (Phynexus Inc.). The purified glycans were dried and resuspended in MiliQ water (20 µl). Initial glycan profiles were obtained by normal-phase HPLC using a TSK-Gel Amide-80 (4.6×250 mm) column run on a Waters 2695 separations module and detected with a Waters 2475 fluorescent detector. The excitation wavelength was set at 320 nm and emission wavelength at 420 nm. Solvent A was 50 mM ammonium formate, pH 4.4. Solvent B was acetonitrile (100%). For initial profiles on HILIC-HPLC, glycans were injected in 80% acetonitrile and run over a 1-h gradient. The system was calibrated using a 2-AB-labelled dextran ladder. Exoglycosidase digestions and WAX-HPLC-fractionated samples were run over a 3-h gradient. Enzymes used were 1 U/ml *Streptococcus pneumoniae* sialidase (Nan1), 1–2 U/ml *Arthrobacter ureafaciens* sialidase (ABS), 1 U/ml *Streptococcus pneumoniae* β-galactosidase, 1 U/ml bovine kidney fucosidase (BKF), 0.1 U/ml *S. pneumoniae* *N*-acetylglucosaminidase (GUH), 1 U/ml Jack Bean mannosidase (JBM). WAX-HPLC was performed using a Vydac 301VHP575 (7.5×50 mm) column (Anachem Ltd, Luton, Bedfordshire, UK). Solvent A was 0.5 M formic acid, pH 9 and solvent B was methanol (10% v/v). 2-AB-labelled glycans were injected in MiliQ water and run over a 30-min gradient. Glycans were separated into differently charged fractions by comparison of their elution times with standard N-linked bovine fetuin carbohydrates. Glycan structures were determined by comparison of retention times, expressed in GU values, with those reported in GlycoBase, in combination with WAX-HPLC, WAX-fractionation and exoglycosidase digestions.

### Mass spectrometry analysis of N-glycans

N-glycans of hCG were analysed by MALDI mass spectrometry following permethylation according to Ciucana and Kerek (Ciucanu and Kerek, 1984). Dried glycans were dissolved in 500 µl DMSO (HPLC grade, Sigma-Aldrich). Powdered sodium hydroxide (99.999%, Sigma-Aldrich) and 100 ml iodo-methane (99.9%, Sigma-Aldrich) were added. Samples were vortexed for 1 h. Samples were placed on ice and 1 ml of water was added followed by 1 ml of HPLC-grade dichloromethane. Sample mixtures were vortexed and the organic phase (containing N-glycans) was removed and saved. The dichloromethane addition was repeated twice and the extracts were pooled. The organic layer was washed with 5× 1 ml HPLC grade H_2_O. Samples were dried prior to MALDI analysis. Permethylated samples were diluted in 75 µl methanol and 25 µl HPLC-grade H_2_O. 1 µl sample was mixed with 1 µl of 2,5-dihydroxybenzoic acid (10 mg/ml in H_2_O:ACN, 1∶1 v/v) on a stainless steel MALDI target plate. Samples were dried and re-crystallised with 0.5 µl ethanol. Permethylated N-glycans were analysed using a Waters MALDI Micro MX mass spectrometer (Manchester, UK) with an ultraviolet 337 nm wavelength nitrogen laser. Instrument settings were as follows: pulse voltage of 2100, suppression voltage of 780 and a laser firing rate of 5 Hz. Spectra processing was performed with Waters MassLynx v4.1 software. Ions were observed as [M+Na]^+^ adducts.

### *In vivo* seminal vesicle weight assay for hCG

The potency of recombinant hCG relative to the International Standard of the hormone or a reference preparation calibrated in international units (IU) was determined under given conditions by measuring the increase in the mass of seminal vesicles (or the prostate gland) of immature rats. The international unit is the activity associated with a stated amount of the International Standard, which consists of a mixture of a freeze-dried extract of hCG from the urine of pregnant women and lactose. The equivalent in international units of the International Standard was established by the World Health Organisation.

Immature male rats of weaning age and of the same strain were used in the study. Animals differed in age by not more than 3 days and had a maximum range in body mass of 10 g. Rats were randomly placed into six groups of at least five animals each, and using one of six littermates per group. Three doses (4 IU, 8 IU and 16 IU) were chosen for the comparison between the reference hCG and recombinant hCG, such that the smallest dose was sufficient to produce a positive response in some of the rats and that the largest dose did not produce a maximal response in any of the rats.

Each rat was injected subcutaneously with a given dose according to its allocated study group on 4 consecutive days at the same time each day. On the fifth day, 24 h after the last injection, rats were killed and the seminal vesicles were harvested. Seminal vesicles were weighed immediately after removing any extraneous fluid and tissue. The precision of the assay was improved by a suitable correction of the organ mass with reference to the total body mass of the animal from which it was taken; an analysis of covariance was used. All animal experiments were performed according to approved guidelines.

## Supplementary Material

Supplementary Material
