# Supplementary material for: Galactosyltransferase 4 is a major control point for glycan branching in N-linked glycosylation
Source: J Cell Sci. 2014 Dec 1;127(23):5014–26. doi: 10.1242/jcs.151878 (PMC4248093; doi:10.1242/jcs.151878)
Supplement: Supplementary Material [file supp_127_23_5014__index.html]

Galactosyltransferase 4 is a major control point for glycan branching in N-linked glycosylation — Supplementary Material 

# Galactosyltransferase 4 is a major control point for glycan branching in N-linked glycosylation

## JCS151878 Supplementary Material

**Files in this Data Supplement:**

- **Supplementary Material**
