# Supplementary material for: Galactosyltransferase 4 is a major control point for glycan branching in N-linked glycosylation
Source: J Cell Sci. 2014 Dec 1;127(23):5014–26. doi: 10.1242/jcs.151878 (PMC4248093; doi:10.1242/jcs.151878)
Supplement: Supplementary Material [file supp_127.23.5014_JCS151878.pdf]

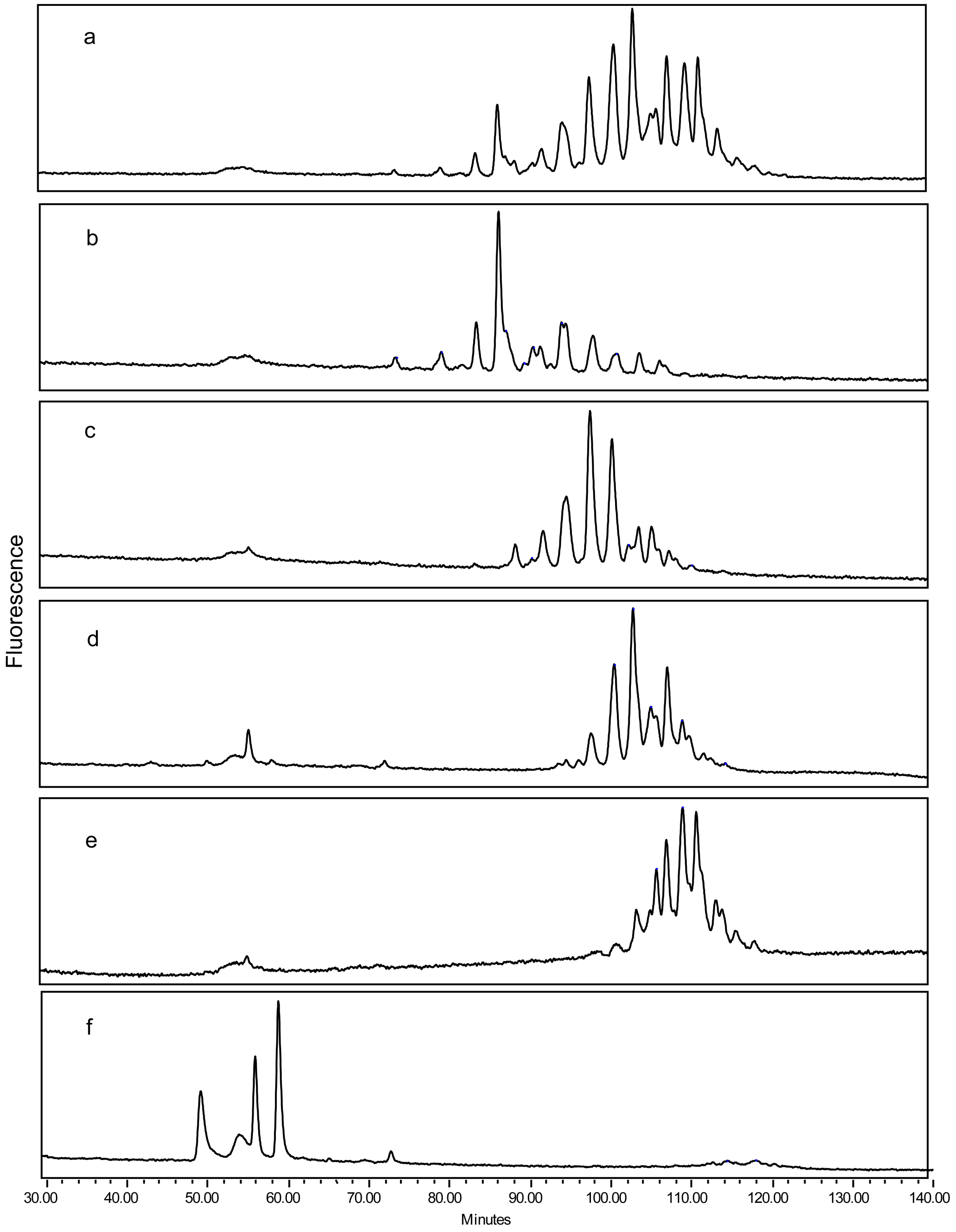

Fig. S1. Tri-sialylated glycan structures are increased in the CHO-GnT4-GalT4 cell line. HILIC analysis of WAX fractionated N-glycans from CHO-GnT4-GalT4 cell line**.** This is a 2-dimensional glycan analysis using WAX in the first dimension and HILIC in the second dimension as a way to deconvolute complex glycan pools for structural assignments. Major increases in tri-antennary structures were observed in this cell line in comparison to hCG purified from CHO cells **a**. Total N-glycan pool; **b**. Neutral fraction; **c**. Mono-sialylated fraction; **d**. Di-sialylated fraction; **e**. Tri-sialylated fraction; **f**. Tetra-sialylated fraction.

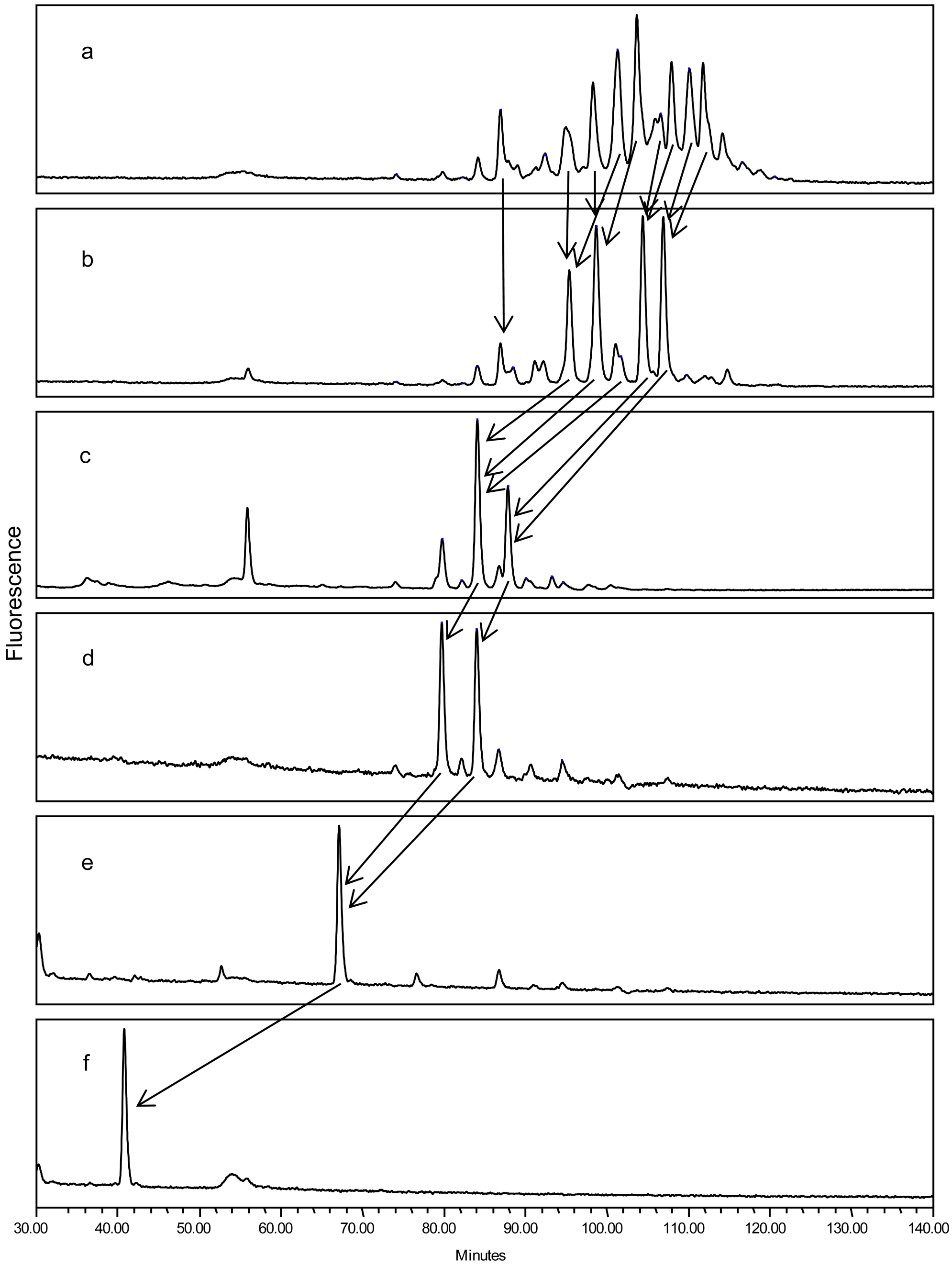

Fig. S2. Exoglycosidase digestions of N-glycans from CHO-GnT4-GalT4. a. Undigested N-glycan pool; **b**. *Arthrobacter ureafaciens* sialidase (ABS) digest, ABS is specific for α2-3/6 linked sialic acids; **c**. ABS + *Streptococcus pneumoniae* (SPG) β-galactosidase digest, SPG is specific for β1,4-linked galactose residues; **d.** ABS + SPG + bovine kidney fucosidase (BKF) digest, BKF is specific to α1,6/2 fucose residues with a preference for core fucoses; **e**. ABS+SPG+BKF+ S. pneumoniae N-acetylglucosaminidase (GUH), GUH is specific for β1,2/4/6-linked GlcNac residues; **f**. ABS+SPG+BKF+GUH+Jack Bean Mannosidase (JBM), JBM is specific for α1-2,6,3 mannose residues. Following digestion HILIC-HPLC was used to analyse the composition of the constituent monosaccharides and to determine individual N-glycan structures.

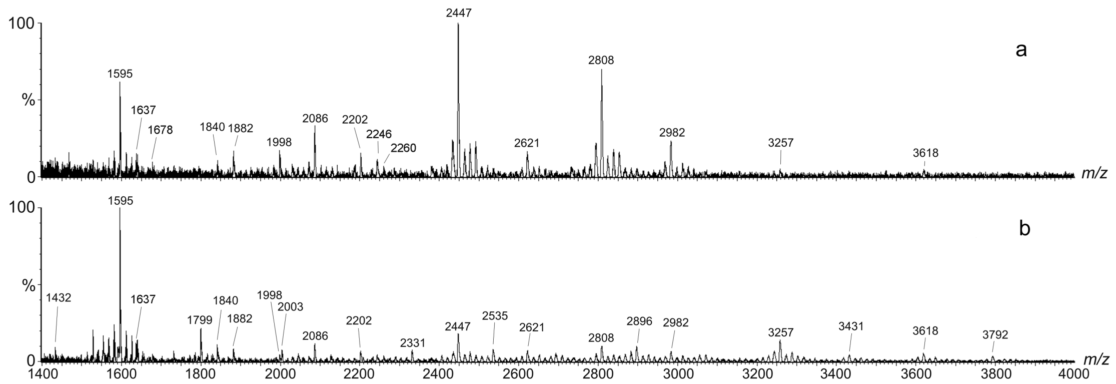

Fig. S3. MALDI-TOF profiles of reduced and permethylated N-glycans from hCG and CHO-GnT4-GalT4

Mass spectrometry was used to verify N-glycan structures identified by HILIC. Permethylated N-glycans were analysed using a Waters MALDI Micro MX mass spectrometer with an ultraviolet 337 nm wavelength nitrogen laser. Spectra processing was performed with Waters MassLynx v4.1 software. Ions were observed as $[M+Na]^+$ adducts. Structures and m/z values are shown in Table SI. **a.** hCG; **b.** CHO-GnT4-GalT4.

Table SI. N-glycan structures identified for hCG purified from CHO-GnT4-GalT4 cell lines.
Structures were determined by a combination of HILIC, WAX-HPLC and exoglycosidase digestions.

Peak	GU Value	Peak Area (%)	Glycan		
			Name	Structure	Observed *m/z* (M+Na)[+]
1	4.94	0.21	A1	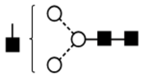	1432.97
2	5.45	0.40	A2	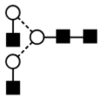	1678.00
		0.15	F(6)A1	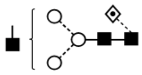	ND
3	5.70	0.27	M4A1	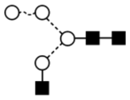	1636.85
4	5.89	0.35	F(6)A2	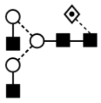	ND
		1.00	A3	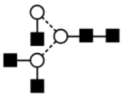	ND
5	6.19	3.97	M5	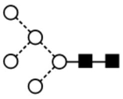	1595.85
6	6.29	0.97	F(6)A3	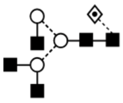	ND
7	6.42	0.87	A1G(4)1S(3)1	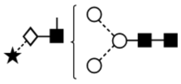	1997.92
8	6.68	1.06	F(6)A2[6]G(4)1	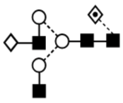	ND
9	6.82	2.49	F(6)A2[3]G(4)1	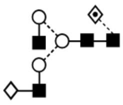	
10	7.13	0.53	F(6)A3G(4)1	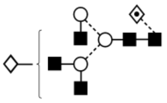	2330.85
		5.50	A2G(4)2	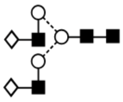	2085.98
11	7.41	0.79	F(6)A2G(4)1S(3)1	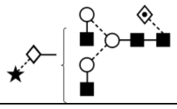	ND

12	7.56	7.57	F(6)A2G(4)2	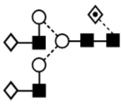	2259.77
13	7.97	9.50	A2G(4)2S(3)1	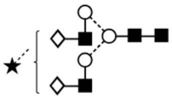	2246.90
		2.73	F(6)A3G(4)1S(3)1	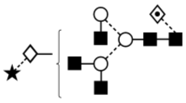	ND
14	8.30	12.44	F(6)A2G(4)2S(3)1	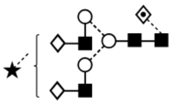	2620.74
		0.89	A3G(4)2S(3)1	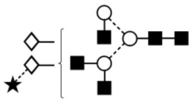	ND
15	8.63	4.00	A2G(4)2S(3)2	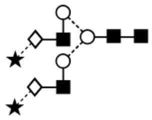	2807.48
		1.15		?	
16	8.73	4.26	F(6)A3G(4)3	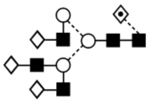	ND
17	8.93		M8	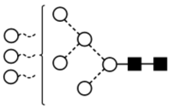	ND
		9.08	F(6)A2G(4)2S(3)2	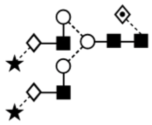	2981.30
18	9.27	4.40	F(6)A3G(4)2S(3)2	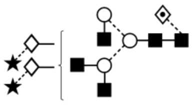	???
		4.4		?	
19	9.54	9.30	F(6)A3G(4)3S(3)1	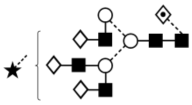	3070.82
		1.36			
20	9.94	4.89	A3G3S2	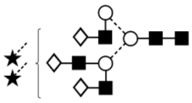	3257.77
21	10.35	2.09	A3G3S3	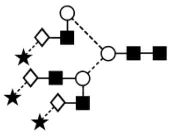	3618.74
22	10.77	1.12	FA3G3S3	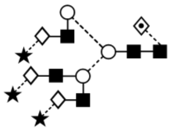	3792.26
